# Treatment-seeking behaviour for febrile illnesses and its implications for malaria control and elimination in Savannakhet Province, Lao PDR (Laos): a mixed method study

**DOI:** 10.1186/s12913-019-4070-9

**Published:** 2019-04-24

**Authors:** Bipin Adhikari, Koukeo Phommasone, Tiengkham Pongvongsa, Palingnaphone Koummarasy, Xayaphone Soundala, Gisela Henriques, Pasathorn Sirithiranont, Daniel M. Parker, Lorenz von Seidlein, Nicholas J. White, Nicholas P. J. Day, Arjen M. Dondorp, Paul N. Newton, Phaik Yeong Cheah, Christopher Pell, Mayfong Mayxay

**Affiliations:** 1 Mahidol-Oxford Tropical Medicine Research Unit, Faculty of Tropical Medicine, Mahidol University, Bangkok, Thailand; 2 Centre for Tropical Medicine and Global Health, Nuffield Department of Medicine, University of Oxford, Oxford, UK; 3 Kellogg College, University of Oxford, Oxford, UK; 4 Lao-Oxford-Mahosot Hospital-Wellcome Trust Research Unit (LOMWRU), Microbiology Laboratory, Mahosot Hospital, Vientiane, Laos; 5 Savannakhet Provincial Health Department, Savannakhet, Province, Laos; 6 Faculty of Infectious and Tropical Diseases, London School of Hygiene and Tropical Medicine, Keppel Street, London, UK; 7 Department of Population Health and Disease Prevention, University of California, Irvine, California, USA; 8 The Ethox Centre, Nuffield Department of Population Health, University of Oxford, Oxford, UK; 9 Centre for Social Science and Global Health, University of Amsterdam, Amsterdam, The Netherlands; 10 Amsterdam Institute for Global Health and Development, Amsterdam, The Netherlands; 11 Institute of Research and Educational Development, University of Health Sciences, Vientiane, Laos

**Keywords:** Health seeking, Malaria, Febrile illness, On the counter, Resistance, Elimination

## Abstract

**Background:**

How people respond to febrile illness is critical to malaria prevention, control, and ultimately elimination. This article explores factors affecting treatment-seeking behaviour for febrile illnesses in a remote area of Lao PDR.

**Methods:**

Household heads or their representatives (*n* = 281) were interviewed using a structured questionnaire. A total of twelve focus group discussions (FGDs) each with eight to ten participants were conducted in four villages. In addition, observations were recorded as field notes (*n* = 130) and were used to collect information on the local context, including the treatment seeking behaviour and the health services.

**Results:**

Almost three-quarters (201/281) of respondents reported fever in past two months. Most (92%, 185/201) sought treatment of which 80% (149/185) sought treatment at a health centre. Geographic proximity to a health centre (AOR = 6.5; CI = 1.74–24.25; for those < 3.5 km versus those > 3.6 km) and previous experience of attending a health centre (AOR = 4.7; CI = 1.2–19.1) were strong predictors of visiting a health centre for febrile symptoms. During FGDs, respondents described seeking treatment from traditional healers and at health centre for mild to moderate illnesses. Respondents also explained how if symptoms, including fever, were severe or persisted after receiving treatment elsewhere, they sought assistance at health centres. Access to local health centres/hospitals was often constrained by a lack of transportation and an ability to meet the direct and indirect costs of a visit.

**Conclusion:**

In Nong District, a rural area bordering Vietnam, people seek care from health centres offering allopathic medicine and from spiritual healers. Decisions about where and when to attend health care depended on their economic status, mobility (distance to the health centre, road conditions, availability of transport), symptoms severity and illness recognition. Current and future malaria control/elimination programmes could benefit from greater collaboration with the locally accessible sources of treatments, such as health volunteers and traditional healers.

## Background

Over recent years, the decline in malaria incidence and malaria-related mortality in the Greater Mekong Sub-region (GMS) has prompted countries in the region to consider the prospect of malaria elimination [1]. As states in the GMS adopt strategies to eliminate malaria, identifying and appropriately managing the remaining cases becomes increasingly critical. In the GMS, the remaining malaria cases together with the burden of parasite reservoir are clustered in hard-to-reach populations, with high mosquito densities, abundance of larval habitats, and human-mosquito interactions, which results from time spent working in forests and the practice of swidden cultivation [2–7].

Laos has made a substantial progress in malaria control and elimination [3]. Nevertheless, country’s southern provinces are still malaria endemic, recording high transmission, with a 2014 annual parasite index (API) of 20.3, in contrast to 7.3 nationally [8]. Five southern provinces (Savannakhet, Saravan, Sekong, Attapeu and Champasak) accounted for 96% of the total malaria cases in 2014 and 36% of the total national population of seven million [3, 8]. As part of the national strategic plan for malaria control and elimination, Laos aims to eliminate *P. falciparum* by 2030 [3, 9, 10].

Strategies piloted for malaria elimination in Laos (and the wider GMS) include the distribution of long lasting insecticide treated bed nets (LLINs), mass drug administrations (MDAs) and strengthening of village malaria/health worker (VMW) networks to ensure appropriate malaria diagnosis and case management [8, 10–13]. Several factors influence the success of these interventions: accessibility particularly in the rainy season; inadequate healthcare infrastructure; poverty; and human behaviour [14–17].

How people respond to febrile illness is critical to malaria prevention, control, and ultimately elimination [18–22]. The many factors that influence behaviour include people’s understanding of illness, their past experiences, and the available methods of diagnosis and treatment [15, 23]. Understanding treatment seeking behaviour and its underpinnings is important for the design of an intervention package and, particularly, the design of the accompanying health messages and the health education tools [21, 24, 25].

In Lao PDR, several studies have explored health seeking behaviour for a number of health conditions, such as maternal and child health [26, 27], sexually transmitted infections [28], respiratory infections [29] and the impacts of available health care infrastructure [30–34]. There is a paucity of published research on health seeking behaviour for febrile illnesses in Laos, specifically focusing on population living in remote, malaria endemic regions. Drawing on qualitative and quantitative data collected prior to a pilot study evaluating targeted malaria elimination (TME), this article examines treatment seeking behaviour for febrile illnesses. The main objective is to explore the patterns and determinants of treatment seeking behaviour for febrile illnesses in four malaria endemic villages of Nong District in Savannakhet province.

### Methods

This mixed-methods study combined a questionnaire-based survey with focused group discussions (FGDs) and structured observations, which were recorded as field notes. The study employed a cross-sectional study design. The combination of methods allowed for triangulation of formal data collection techniques (questionnaire and FGDs) with recording information on informal behaviours and utterance in real-life situations. The findings from the questionnaire-based survey were explored using three qualitative data collection techniques. This provided further information underlying the responses provided in the survey.

The questionnaire-based survey enabled the research team to collect community-wide data and elicit the opinions of all the households in the targeted communities. Such a community-wide approach was taken in the context of MDA, which targeted all community members regardless of malaria infection status. In the villages selected for this intervention (which also included blood tests over three consecutive months), it was critical to explore thoroughly the treatment seeking behaviour for the febrile illnesses and malaria so as to design a tailored community engagement to maximize the uptake of MDA [21, 24]. FGDs were conducted about a week after the completion of the questionnaire survey to explore the reasons that underpinned responses to the questionnaire survey. Observational field notes were collected throughout the study period. Observations of behaviours and utterances in the communities where MDA was conducted were used to provide wider information on the local social and cultural context relevant to malaria, febrile illnesses, preventive measures, treatment seeking and the wider healthcare situation, which are not dictated by the themes and topics of the questionnaire survey and FGDs. The observations were recorded in field notes and typed up for analysis.

The design of the questionnaire was based on a review of literature discussed below. The themes addressed in the FGD guide were designed to reflect the topics covered in the questionnaire.

### Study context

**Fig. 1:**
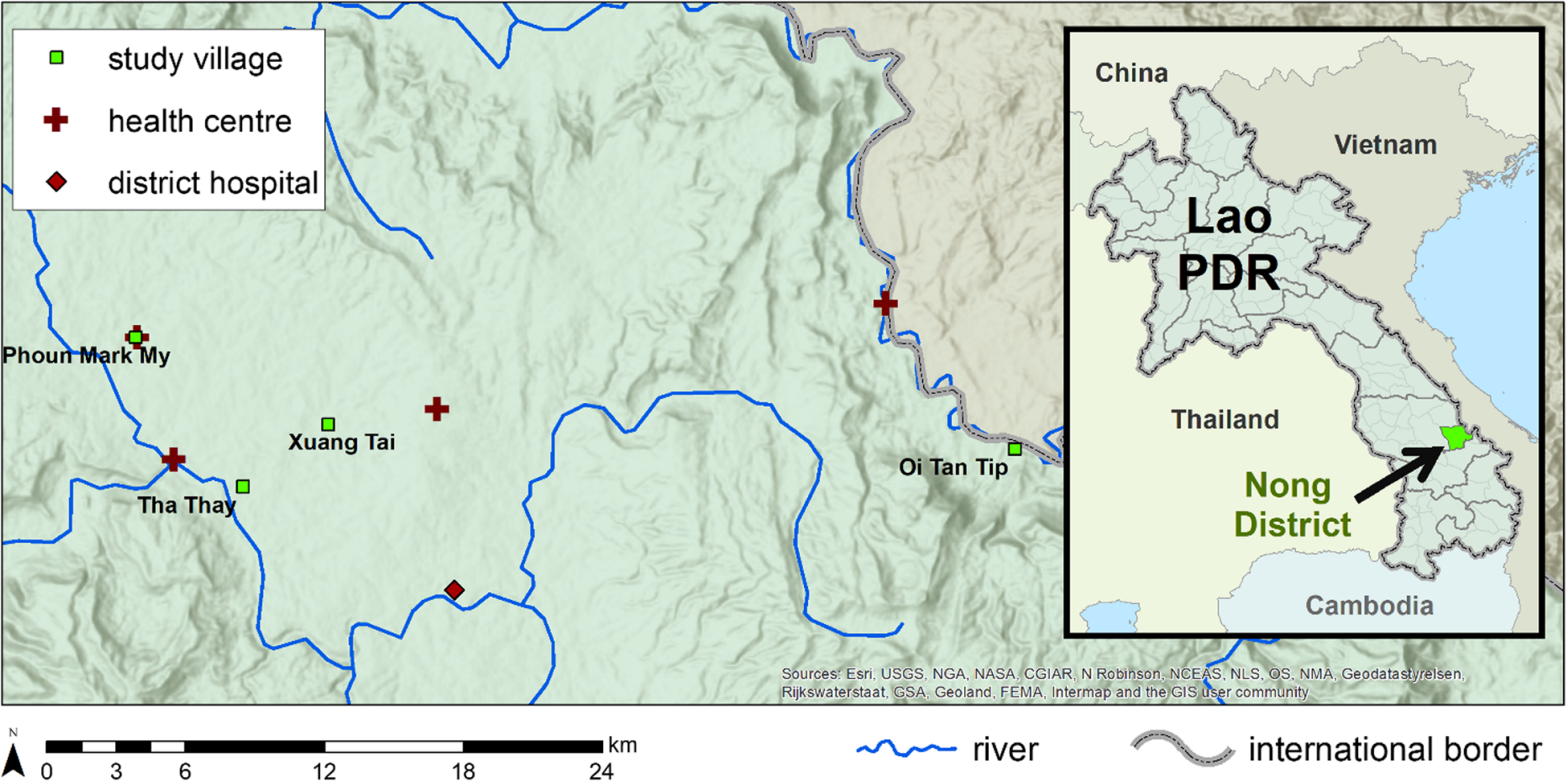
Study villages, health centres and a hospital in Nong district, Savannakhet province, Lao PDR. The map in Fig. 1 was created for this manuscript by one of the co-authors (DMP) using ArcMap 10.5.1 and GPS coordinates taken from the field locations

The questionnaire-based survey and FGDs were conducted between November and December 2015, prior to the start of the TME pilot study [3]. Based on existing malaria endemicity data, two districts in Savannakhet (Nong and Thapangthong) were surveyed using ultrasensitive quantitative PCR (uPCR) and four villages in Nong District were ultimately selected for TME based on the high prevalence of asymptomatic malaria (Fig. 1) [3]. The current study was a sub-study of the main TME pilot and data were collected in the TME villages.

### Study participants and questionnaire

Villages comprised population sizes between 300 and 500 residents: Oi Tantip (OTP = 512), Phoun Mak Mee (PMM = 480), Thate (TT = 526) and Xuang Tai (XT = 371) [20]. The study population consisted of one member, preferably the household head, of every household in the four TME villages. There were total of 281 households in 4 villages (OTP = 65, PMM = 74, TT = 82 and XT = 60). Each respondent, representing a household, was interviewed using a structured questionnaire that consisted of questions on socio-demographic characteristics and health seeking behaviour in response to febrile illness.

Two Laotian social scientists interviewed respondents at their household after obtaining written informed consent, mostly in the Lao language. For a few respondents who could not speak the Lao language, a bilingual local study coordinator translated on site to note down the responses by interviewers. A household head or any other representative of house above 18 years old were selected for the study. If representatives of the household were not present during the survey, they were followed up during the consecutive days. None of the household representatives refused to participate. Respondents representing a total of two households were away and interviewed once they returned. Representatives from all households within four selected villages were ultimately interviewed. Interviews lasted between 30 and 45 min.

The questionnaire was drafted in English with the help of two Laotian social scientists, translated into Lao Language and back translated to English to ensure accuracy and validity. For this study, the questionnaire consisted of three parts: I general information about the interview, interviewer and interviewee; II socio-demographic characteristics, such as age, sex, religion and marital status; and III health seeking behaviour [9]. Socio-economic variables were designed after reviewing the past studies from various regions within Laos [28, 29, 34], Vietnam [35], Cambodia [36], Thai-Myanmar border [37] and The Gambia [37, 38]. In addition, questions on socio-economic status have been recently used in sub-studies from the TME villages in Laos [9, 12]. Questions on health seeking behaviour for febrile illness were constructed after reviewing the past studies, conducted in Laos [28, 29], Cambodia [36], Uganda [39] and Ghana [40] and further adapted to suit the local cultural context.

All data are drawn from participants’ responses apart from the approximate distance between household and the health centre, which were estimated by the interviewers. Based on the pre-testing of the questionnaire amongst the experts, respondents around Mahosot Hospital and the households around Nong district, history of fever in last two months were (chosen) asked to minimize recall bias. The question on health seeking behaviour was: *“if you have fever, where do you usually go for treatment first?”* Responses that mentioned allopathic health care services, such as health centre/health post, private pharmacies, mobile drug vendors, village health volunteers, district, provincial and central hospitals were categorized as “Allopathic health services” and other services such as traditional healers, including spiritual healers and sorcerers were categorized as “non-allopathic health services”.

Two social scientists pre-tested the data collection tools, as administering it first to the site PI and two authors and later to 15 volunteer participants around Mahosot Hospital in Vientiane. Further pre-testing was carried out in Nong District with six households. Ambiguities were corrected and simplified.

### Focus group discussions and field notes

Twelve FGDs were conducted with a total of 100 participants (with 8–10 participants per each FGD) from four villages and their sub-villages (Table 1). There were a total of six villages/sub-villages. Two focus group discussions (one each for males and females) were conducted in each of these six villages/sub-villages. In each village/sub-village, a village head was asked to invite eight to 10 community members to participate in a FGD. However, the number of participants attended was always higher than that required. Considering the sensitivity around turning them away in light of their enthusiasm, a random sample of eight to 10 adult participants (above 18 years and consenting to participate) were selected using a lottery method during village meetings. Male and female participants were interviewed in separate groups by a male and a female interviewer respectively. Aware of the local social and cultural context, whereby patriarchy is strong and there is a tendency for conformism among females when mixed with males groups, male and female FGDs were conducted separately [12, 41].

**Table 1:** Socio-demographic characteristics of FGD Participants (*n* = 100)

	Total	Village: Number (%)					
Characteristics	Number (%)	Oi	Tantip	PMM	Keng and Appok	TT main	XT
Age Group							
≤ 35 years	51 (51)	7 (13.7)	12 (23.5)	6 (11.8)	13 (25.5)	7 (13.7)	6 (11.8)
> 36 years	49 (49)	9 (18.4)	4 (8.2)	12 (24.5)	5 (10.2)	9 (18.4)	10 (20.4)
Mean = 37.58 ± 12.71; Range = 18–80 years							
Sex							
Male	52 (52)	8 (15.4)	8 (15.4)	10 (19.2)	10 (19.2)	8 (15.4)	8 (15.4)
Female	48 (48)	8 (16.7)	8 (16.7)	8 (16.7)	8 (16.7)	8 (16.7)	8 (16.7)
Occupation							
Farmer	100 (100)	16 (16)	16 (16)	18 (18)	18 (18)	18 (18)	16 (16)
Education Groups							
Not attended school	82 (82)	12 (14.6)	10 (12.2)	13 (15.9)	17 (20.7)	14 (17.1)	16 (19.5)
1–5 years	16 (16)	4 (25)	5 (31.3)	5 (31.3)	1 (6.3)	1 (6.3)	0 (0)
> 6 years	2 (2)	0	1 (50)	0	0	1 (50)	0

The discussions took place in the home of one of the villagers. The interviewers made use of an open-ended questionnaire guide (FGD guide) developed by the study team which included sections I and II. The section I explored the “treatment seeking behaviour for febrile illnesses and malaria”, and section II explored “knowledge and attitude related to malaria, preventive measures and mass drug administration” [9]. For the purpose of this study, section I was utilized to focus on health seeking behaviour on broad themes such as *“When and how do you decide to go for treatment when you are sick?”* Each FGD lasted for about 60 to 120 min. The discussions were conducted in *Pasha Lao* and translated into the local *Lao Theung* language on site. In this article, *“Lao Theung”* refers to language spoken by members of people who reside in these villages. All respondents gave written consent to record and analyse their replies. The discussions were recorded, transcribed and translated into English by the social scientists who conducted the FGDs.

In addition to FGDs, field notes were collected based on the observation of the TME- related activities, including the local health infrastructure, road conditions, transportation means and common health practices. Field notes contained the location, subject of the notes, date and descriptions of the issues in question (for example: health services, traditional practices) based on the formal/informal discussions, meetings and interviews. Field notes were taken by BA, XS and PK in English with on-site translation where necessary. Field notes were collected each time study team made a visit to the study villages and each field note represented a day’s observation. A total of 130 field notes were collected from September 2015 to August 2016. Observations were made at every visit to the villages and these were recorded as field notes.

### Data management and analysis

The questionnaire responses were entered into the Open Data Kit application on a smartphone in English (ODK; Available online: https://opendatakit.org/). Two social scientists conducted the interviews using two smartphones with on-site translation from either a local study coordinator or a bilingual village volunteer (fluent in *Pasha Lao* and *Pasha Lao Theung*) and were recorded in English in ODK. All collected data were extracted into Excel sheet (Microsoft Excel 2013) at Mahidol-Oxford Tropical Medicine Research Unit where data manager made cross-checks for any aberrant and outlying data. Data were analyzed in IBM SPSS Statistics for Windows, Version 24.0, Armonk, NY: IBM Corp.

Descriptive analysis, such as frequencies, percentages, were calculated and cross tabulated with health seeking behavior for febrile illness categorized into attending: 1. Allopathic health services and 2. non-allopathic health services, using Chi squared and fisher exact test wherever appropriate. An association was considered significant at *p* value ≤0.05. Similarly, bivariate and multivariate analysis were conducted using logistic regression models to calculate odds ratio.

Based on the statistical significance (p value ≤0.05) and relevance to the research question, variables were dichotomized as the presence of certain characteristics as 1 and their absence as 0. These variables were analyzed using a multi-variate logistic regression model. At first, bivariate analysis was conducted with the dependent variable (health seeking behaviour categorized as attending allopathic health services as “1” and non-allopathic health services as “0”) to calculate crude odds ratio. In the multi-variate model, age and sex were controlled for in the model to calculate the adjusted odds ratio.

Translated and transcribed FGD transcripts together with field notes were analysed using the qualitative data analysis software QSR NVivo 11. All transcripts were coded line-by-line using pre-set themes (deductive approach) as nodes. Further nodes were added for emerging themes (inductive approach). Data were coded by two researchers (BA and CP) independently based on the pre-set themes which used to guide the overall study. Final codes for the study were decided based on the consensus amongst the researchers and relevance to the objective of this study. All the nodes were analysed for the pattern and the content to answer the research question. The quotes presented in this study are the typically expressed concerns and are not “occasional” or “exceptional”.

## Results

### Socio-demographic and household characteristics of participants

A total of 100 participants from villages and sub-villages were included in 12 FGDs which had a similar proportion of male (52%) and female (48%) participants. Their mean age was 37 years, 82% did not attend school and all were farmers (Table 1).

Almost all respondents (96%, 269/281) were from minority ethnic groups that consisted of *Mangkong* = 200 (71.2%), *Tree* = 64 (22.8%), *Phu Thai* = 6 (2.1%), *Ta Oi* = 3 (1.1%), and *Ka Tarng* = 1 (0.4%) and rest of the other included *Vietnamese* = 4 (1.4%), and *Lao Loum* = 3 (1.1%). The majority **(**76%; 201/281) were male, illiterate 76% (214/281) and 73% (204/281) never attended formal education. Respondents who had attended a school and were of a high socio-economic status (25/27; 93% from monthly income of 240 USD attended health centre) compared to respondents from low (< 60 USD; only 68%; 131/192 attended health centre) socio-economic status (*p* = 0.007) were more likely to attend allopathic health services than respondents who had not attended school and were from a lower socio-economic status (*p* = 0.034; Table 2).

**Table 2:** Treatment seeking behaviour for febrile illnesses in relation to socio-economic characteristics of the participants (n = 281)

Characteristics	Total (n = 281)	Allopathic health services (*n* = 200)	Non-allopathic health services (*n* = 81)	*p*-value
	Number (%)	Number (%)	Number (%)	
Villages				
OTP	65 (23.1)	52 (26)	13 (16)	0.306
PMM	74 (26.3)	50 (25)	24 (29.6)	
TT	82 (29.2)	55 (27.5)	27 (33.3)	
XT	60 (21.4)	43 (21.5)	17 (21)	
Respondent status				
Family head	188 (66.9)	139 (69.5)	49 (60.5)	0.309
Wife of family head	70 (24.9)	45 (64.3)	25 (30.9)	
Other	23 (8.2)	16 (8)	7 (8.6)	
Age Group				
≤ 30 years	91 (32.4)	63 (31.5)	28 (34.6)	0.869
31–50 years	138 (49.1)	99 (49.5)	39 (48.1)	
≥ 51 years	52 (18.5)	38 (19)	14 (17.3)	
Sex				
Male	201 (71.5)	149 (74.5)	52 (64.2)	0.083
Female	80 (28.5)	51 (25.5)	29 (35.8)	
Ethnicity^a^				
Laothung	269 (95.7)	192 (96)	77 (95.1)	0.472
Other	12 (4.3)	8 (4)	4 (4.9)	
Religion				
Buddhist	9 (3.2)	8 (4)	1 (1.2)	0.213
Animist	272 (96.8)	192 (96)	80 (98.8)	
Marital status				
In relationship	262 (93.2)	186 (93)	76 (93.8)	0.518
Not in relationship	19 (6.8)	14 (7)	5 (6.2)	
Literacy				
Literate	67 (23.8)	54 (27)	13 (16)	0.051
Illiterate	214 (76.2)	146 (73)	68 (84)	
Level of Education				
Not attended school	204 (72.6)	138 (69)	66 (81.5)	0.034
Attended school	77 (27.4)	62 (31)	15 (18.5)	
Occupation				
Farmer	254 (90.4)	183 (91.5)	71 (87.7)	0.322
Other	27 (9.6)	17 (8.5)	10 (12.3)	
Monthly Income				
≤ 500,000 kip (≤60 USD)	192 (68.3)	131 (65.5)	61 (75.3)	0.007
500,001 to 2,000,000 kip (60 to 240 USD)	41 (14.6)	33 (16.5)	8 (9.9)	
≥ 2,000,001 kip (≥240 USD)	27 (9.6)	25 (12.5)	2 (2.5)	
Don’t know/NA	21 (7.5)	11 (52.4)	10 (12.3)	
Daily Expense				
≤ 1000 kip (≤0.12 USD)	99 (35.2)	72 (36)	27 (33.3)	0.289
1001 to 10,000 kip (0.12 to 1.18 USD)	120 (42.7)	80 (40)	40 (49.4)	
≥ 10,000 kip (≥1.18 USD)	62 (22.1)	48 (24)	14 (17.3)	
Is the income enough to sustain living?				
Yes	82 (29.2)	52 (26)	30 (37)	0.047
No	198 (70.5)	148 (74)	50 (61.7)	
Not sure/Don’t know	1 (0.4)	0	1 (1.2)	

^a^*Ka Tarng* = 1 (0.4%), *Lao Loum* = 3 (1.1%), *Mangkong* = 200 (71.2%), *Phu Thai* = 6 (2.1%), *Ta Oi* = 3 (1.1%), *Tree* = 64 (22.8%), *Vietnamese* = 4 (1.4%)

Respondents who reported attending allopathic health services, were more likely to own a tractor (*p* = 0.013) and lived further from the forest than respondents who owned no tractors or lived closer to the forest (p = 0.007; Table 3). Most households (275/281; 98%) owned agricultural land and more than half (153/281; 54%) owned a motorbike (Table 4).

**Table 3:** Treatment seeking behaviour for febrile illnesses in relation to household characteristics of the participants (*n* = 281)

Characteristics	Total (n = 281)	Allopathic health services (*n* = 200)	Non-allopathic health services (*n* = 81)	*p*-value
	Number (%)	Number (%)	Number (%)	
What do you own?^a^				
House	281 (100)	200 (71.2)	81 (28.8)	NA
Land	275 (97.9)	196 (71.3)	79 (28.7)	0.556
Motorbike	153 (54.4)	106 (69.3)	47 (30.7)	0.263
Bicycle	7 (2.5)	3 (42.9)	4 (57.1)	0.109
Tractor	51 (18.1)	29 (56.9)	22 (43.1)	0.013
Cars	4 (1.4)	3 (75)	1 (25)	0.673
Cattle	118 (42)	87 (73.7)	31 (26.3)	0.421
TV	53 (18.9)	38 (71.7)	15 (28.3)	0.926
Radio	11 (3.9)	5 (45.5)	6 (54.5)	0.062
Phones	122 (43.4)	91 (74.6)	31 (25.4)	0.165
What are your walls of your home made from?^a^				
Bamboo	126 (44.8)	94 (74.6)	32 (25.4)	0.253
Wood	189 (67.3)	128 (67.7)	61 (32.3)	0.067
Concrete	1 (0.4)	0	1	0.288
Plastic	10 (3.6)	7 (70)	3 (30)	0.588
Metal	8 (2.8)	3 (37.5)	5 (62.5)	0.047
What is the roof of your home made from?^a^				
Bamboo	21 (7.5)	16 (76.2)	5 (23.8)	0.402
Wood	4 (1.4)	3 (75)	1 (25)	0.673
Plastic	5 (1.8)	5 (100)	0	0.18
Metal	212 (75.4)	147 (69.3)	65 (30.7)	0.234
Shingles	53 (18.9)	41 (77.4)	12 (22.6)	0.175
What is the floor of your home made from?^a^				
Bamboo	44 (15.7)	31 (70.5)	13 (29.5)	0.909
Wood	251 (89.3)	181 (72.1)	70 (27.9)	0.212
Mud	2 (0.7)	2 (100)	0	0.506
Concrete	8 (2.8)	3 (37.5)	5 (62.5)	0.047
Do you have toilet facility at home?				
Yes	22 (7.8)	14 (7)	8 (9.9)	0.279
No	259 (92.2)	186 (93)	73 (90.1)	
Did you migrate from any other village?				
Yes	61 (21.7)	43 (21.5)	18 (22.2)	0.894
No	220 (78.3)	157 (78.5)	63 (77.8)	
If Yes how long have you been living here?				
≤ 10 years	28 (45.9)	18 (41.9)	10 (55.6)	0.617
11–20 years	17 (27.9)	13 (30.2)	4 (22.2)	
≥ 21 years	16 (26.2)	12 (27.9)	4 (22.2)	
How far is the forest from your house in km?				
≤ 1 km	97 (34.5)	57 (28.5)	40 (49.4)	0.007
1.1 to 2 km	76 (27)	62 (31)	14 (17.3)	
≥ 2.1 km	49 (17.4)	37 (18.5)	12 (14.8)	
NA	59 (21)	44 (22)	15 (18.5)	
How far is the forest from your house in min?				
≤ 7 min	19 (34.5)	16 (39)	3 (21.4)	0.442
7.1 to 60 min	21 (38.2)	14 (34.1)	7 (50)	
≥ 61 min	15 (27.3)	11 (26.8)	4 (28.6)	
How far is your rice field from your house in km?				
≤ 1 km	75 (26.7)	56 (28)	19 (23.5)	0.331
1.1 to 2 km	84 (29.9)	64 (32)	20 (24.7)	
≥ 2.1 km	56 (19.9)	36 (18)	20 (24.7)	
NA/Not own Land	66 (23.5)	44 (22)	22 (27.2)	
How far is your rice field from your house in Min?				
≤ 20 min	18 (33.3)	13 (35.1)	5 (29.4)	0.577
21 to 60 min	30 (55.6)	21 (56.8)	9 (52.9)	
≥ 61 min	6 (11.1)	3 (8.1)	3 (17.6)	
How often do you go to forest?				
Everyday	171 (60.9)	126 (63)	45 (55.6)	0.441
Every Alternate Day	68 (24.2)	47 (23.5)	21 (30.9)	
≥ Weekly	42 (14.9)	27 (13.5)	15 (18.5)	

^a^Includes only “yes” answers and since multiple answers were possible, percentage do not add to 100

**Table 4:** Treatment seeking behaviour for febrile illnesses in relation to health center and past behaviour (n = 281)

Characteristics	Total (n = 281)	Allopathic health services (n = 200)	Non-allopathic health services (n = 81)	*p*-value
	Number (%)	Number (%)	Number (%)	
Have you ever become sick or had fever in the past 2 months?				
Yes	201 (71.5)	143 (71.5)	58 (71.6)	0.986
No	80 (28.5)	57 (28.5)	23 (28.4)	
If Yes, did you seek treatment? (*n* = 201)				
Yes	185 (92)	134 (93.7)	51 (87.9)	0.14
No	16 (8)	9 (6.3)	7 (12.1)	
If Yes, where did you first seek treatment? (*n* = 185)				
Self-treatment				
No	184 (99.5)	134 (100)	50 (98)	0.276
Yes	1 (0.5)	0	1 (2)	
Sorcerer				
No	137 (74.1)	123 (91.8)	14 (27.5)	< 0.001
Yes	48 (25.9)	11 (8.2)	37 (72.5)	
Health center				
No	36 (19.5)	14 (10.4)	22 (43.1)	< 0.001
Yes	149 (80.5)	120 (89.6)	29 (56.9)	
Other				
No	167 (90.3)	121 (90.3)	46 (90.2)	0.588
Yes	18 (9.7)	13 (9.7)	5 (9.8)	
Which do you prefer more between?				
Lao Traditional Medicine	4 (1.4)	3 (1.5)	1 (1.2)	0.023
Allopathic Health Services	274 (97.5)	197 (98.5)	77 (95.1)	
NA	3 (1.1)	0	3 (3.7)	
How far is the nearest health center from your home in km?				
≤ 3.5 km	94 (33.5)	84 (42)	10 (12.3)	< 0.001
3.6 to 12 km	99 (35.2)	67 (33.5)	32 (39.5)	
≥ 12.1 km	88 (31.3)	49 (24.5)	39 (48.1)	
How far is the nearest health center from your home in min?				
≤ 30 min	49 (63.6)	22 (52.4)	27 (77.1)	0.065
≥ 31 min	26 (33.8)	19 (45.2)	7 (20)	
NA	2 (2.6)	1 (2.4)	1 (2.9)	
How do you get to the health center/hospital?				
By walk	138 (49.1)	97 (48.5)	41 (50.6)	0.002
By some means	136 (48.4)	102 (51)	34 (42)	
NA	7 (2.5)	1 (0.5)	6 (7.4)	
Is the road to nearest health center in good condition?				
Yes	214 (76.2)	157 (78.5)	57 (70.4)	0.002
No	62 (22.1)	43 (21.5)	19 (23.5)	
Don’t know	5 (1.8)	0	5 (6.2)	
Are you satisfied with your nearest health center?				
Yes	261 (92.9)	192 (96)	69 (85.2)	< 0.001
No	8 (2.8)	6 (3)	2 (2.5)	
Don’t know	12 (4.3)	2 (1)	10 (12.3)	
Can you get medicine on the counter?				
Yes	85 (30.2)	70 (35)	15 (18.5)	< 0.001
No	188 (66.9)	129 (64.5)	59 (72.8)	
Don’t know	8 (2.8)	1 (0.5)	7 (8.6)	
Do you prefer on the counter medicine?				
Yes	23 (8.2)	12 (6)	11 (13.6)	0.001
No	246 (87.5)	184 (92)	62 (76.5)	
Don’t know	12 (4.3)	4 (2)	8 (9.9)	
If Yes, Why?				
Because it is easy				
No	5 (21.7)	5 (41.7)	0	0.024
Yes	18 (78.3)	7 (58.3)	11 (100)	
I don’t like to go to health center				
No	22 (95.7)	11 (91.7)	11 (100)	0.522
Yes	1 (4.3)	1 (8.3)	0	
It is faster				
No	15 (65.2)	9 (75)	6 (54.5)	0.278
Yes	8 (34.8)	3 (25)	5 (45.5)	
It is cheaper				
No	22 (95.7)	12 (100)	10 (90.9)	0.478
Yes	1 (4.3)	0	1 (9.1)	
Because doctors do not prescribe				
No	21 (91.3)	10 (83.3)	11 (100)	0.261
Yes	2 (8.7)	2 (16.7)	0	
Other				
No	22 (95.7)	11 (91.7)	11 (100)	0.522
Yes	1 (4.3)	1 (8.3)	0	
NA				
No	21 (91.3)	10 (83.3)	11 (100)	0.261
Yes	2 (8.7)	2 (16.7)	0	

### Health care infrastructure in study villages

Based on the observations captured in field notes, all four villages; Oi Tan Tip (OTP), Phoun mak mee (PMM), Tha Thay (TT) and Xuang Tai (XT) were home to members of the *Lao Theung* ethnic minorities (Fig. 1). In this article, “*Lao Theung*” refers to residents of the study villages, a group composed of various ethnic groups, mostly *Tree* and *Mangkong* and are described below*.* OTP consisted two sub-villages Oi and Tantip both located at the border of Vietnam separated by the river. There is no health centre in OTP and primary health care was provided by village health volunteers. The nearest health centre was 10 km away. In PMM there is a health centre within the confines of the village and the houses were clustered into a single village. Three sub-villages (Appok, TT main and TT Geng) comprised TT and the nearest health centre was about 3 km away. In XT, there were two sub-villages (Xung Ngai and Xuang Gang) and the nearest health centre was about 5 km away. Amongst the villages, OTP was farthest from the district hospital (approximate distance to district hospital for OTP ≥ 40 km, PMM ≥ 25 km, XT ≥ 10 km and TT was ≥18 km). In all these government health facilities, diagnosis for malaria and treatment are available for free. Apart from the facilities offering primarily allopathic services, traditional healing embedded in spiritual practices, referred in this study as “Animist religion” was available to residents.

Data recorded from observations highlighted how traditional healing consisted of mostly informal spiritual practices, specific to this religion. In general, traditional healers could be divided into spiritual healers and sorcerers. Spiritual healers were consulted for minor illnesses and were easily found. For more severe and chronic illnesses, such as repeated fever episodes or persistent weight-loss, sorcerers practising witchcraft were consulted. The sorcery healing aims to pacify disease-causing spirits by sacrificing animals such as pigs, chicken and cows. In all villages and sub-villages, traditional practitioners were respected and used alongside the allopathic health centres.

Community members also purchased over-the-counter medicines from authorized or unauthorized pharmacies, including, in Nong District, mobile drug vendors who visit from Vietnam. These drugs were commonly bought for minor complaints, such as headache, common cold and lower back pain. They are cheap treatment options compared to costs of visiting a health centre.

### Factors affecting treatment seeking at allopathic health facilities and general patterns of health seeking behaviour

Almost three-quarters of participants (72%, 201/281) reported having fallen sick or had fever in the past two months and more than 90% of these participants (185/201) sought treatment. Half of the respondents (49.1% 138/281) walked to the nearest health centre. A minority (8%, 16/201) who did not seek treatment responded that they were either self-cured, took medicine from village health volunteers or from families or friends. Among those who sought treatment, 71% sought treatment at allopathic health services (200/281). Past health care seeking practices, such as visiting a health centre (*p* < 0.001), were associated with the reported preference for allopathic health services. Other factors such as distance to health centre (p < 0.001), a good road to the health centre (*p* = 0.002), and satisfaction with the services from health centre (p < 0.001) were all associated with increased attendance at allopathic health services (Table 4).

Bivariate and multivariate logistic regression were conducted to explore independent factors associated with attendance at an allopathic health services. Respondents who belonged to the lowest income group (AOR = 0.16; CI = 003 to 0.76; *p* = 0.02), who had sought assistance from a spiritual healer or witch in the past (AOR = 0.02; CI = 0.005 to 0.08; p < 0.001) and who preferred medicine over-the-counter (AOR = 0.04; CI = 0.008 to 0.22; p < 0.001) were less likely to attend allopathic health services. In contrast, participants who attended a health centre in the past (AOR = 4.69; CI = 1.15 to 19.06; *p* = 0.03) and who stayed nearest to the health centre (AOR = 6.5; CI = 1.74 to 24.25; *p* = 0.005) were more likely to attend an allopathic health services (Table 5).

**Table 5:** Logistic regression analysis to identify independently significant variables associated with treatment seeking at Allopathic health services for febrile illnesses

Covariates	Responses		Univariate Analysis	*p*-value	Multivariate analysis	*p*-value
	Yes	No	Crude OR (95% CI)		AOR^a^ (95% CI)	
	Number (%)	Number (%)				
Attended school	77 (27.4)	204 (72.6)	1.97 (1.0 to 3.73)	0.036	0.53 (0.14 to 2.02)	0.35
Lowest income group	192 (68.3)	89 (31.7)	0.62 (0.34 to 1.15)	0.11	0.16 (0.03 to 0.76)	0.02
Perceived enough income	82 (29.2)	199 (70.8)	0.59 (0.34 to 1.03)	0.067	1.1 (0.25 to 4.76)	0.89
Owns Tractor	51 (18.1)	230 (81.9)	0.45 (0.24 to 0.85)	0.014	0.29 (0.05 to 1.53)	0.14
House from forest ≥2.1 km	49 (17.4)	232 (82.6)	1.3 (0.64 to 2.65)	0.46	2.88 (0.71 to 11.67)	0.13
Went to Sorcerer	48 (25.9)	137 (74.1)	0.03 (0.01 to 0.081)	< 0.001	0.02 (0.005 to 0.08)	< 0.001
Went to health center	149 (80.5)	36 (19.5)	6.5 (2.97 to 14.23)	< 0.001	4.69 (1.15 to 19.06)	0.03
Prefer allopathic medicine	274 (97.5)	7 (2.5)	3.41 (0.74 to 15.59)	0.11	0.09 (0.004 to 2.09)	0.13
Health center is nearest (< 3.5 km)	94 (33.5)	187 (66.5)	5.14 (2.5 to 10.55)	< 0.001	6.5 (1.74 to 24.25)	0.005
Road to health center is good	214 (76.2)	67 (23.8)	1.53 (0.85 to 2.75)	0.14	0.56 (0.16 to 1.95)	0.36
Satisfied with the health center	261 (92.9)	20 (7.1)	4.17 (1.63 to 10.64)	0.003	2.19 (0.32 to 14.61)	0.41
Can get medicine on the counter	85 (30.2)	196 (69.8)	2.36 (1.26 to 4.45)	0.007	1.22 (0.26 to 5.72)	0.79
Prefer medicine on the counter	23 (8.2)	258 (91.8)	0.4 (0.17 to 0.96)	0.041	0.04 (0.008 to 0.22)	< 0.001

^a^Adjusted for variables age and sex

**Fig. 2:**
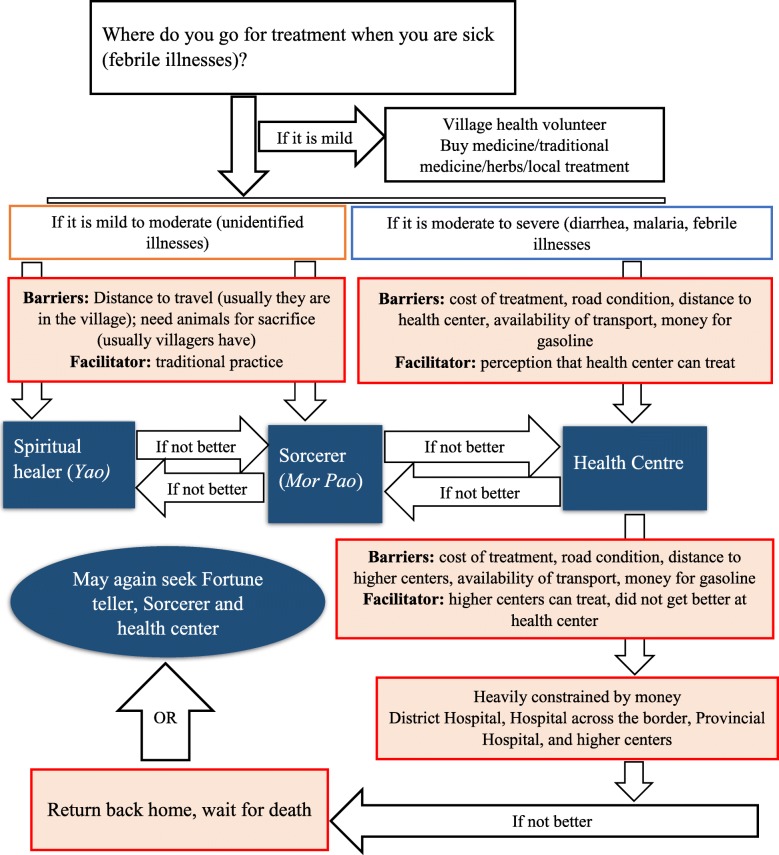
Schematic map of treatment seeking behavior of villagers

These findings were further complemented by the qualitative data from focus group discussions and observational field notes. In line with quantitative data, qualitative methods bolstered how the barriers (cost of treatment, distance to the health centres and available means of transport) were embedded in their patterns of treatment seeking at various places (Fig. 2).

Villagers described a mental map where to seek care, depending upon the type of illness, and constraints to accessing the facility of choice, such as finances, distance to the facilities and the means of transportation. Respondents often reiterated the constraints, mostly the lack of cash money in these villages. The domestic cattle owned by the household were often the source of cash money and during emergency, depending upon the availability of cattle and the buyer, domestic cattle were sold for the cash. In addition to a high poverty in these villages, lack of cash money during emergency was a main barrier for visiting health centres.

*I: Are there any reasons why someone in this community would not seek treatment?*

*FGD1S1: If the [villagers] do not have money [they will not go]. If they have, they will go.*

*FGD1S2: We do not have money [to go for the treatment].*

*(All others agreed).*

*I: Are there reasons why someone in this village would not seek treatment (for fever)?*

*FGD1S2: No money.*

*I: What about the transportation?*

*FGD1S2: Even if you have motorbike, if you don’t (have) money for gasoline, you cannot go.*

FGD with eight female participants in “Oi” sub-village of OTP.

For mild illnesses (e.g. cold), community members often sought assistance from village health volunteers in addition to buying medicine over-the-counter or taking traditional remedies or herbs, which are prevalent in the villages (Fig. 2). For mild to moderate illnesses, respondents were likely to first attend traditional healers and then allopathic health services (health centres). If villagers recognized specific diseases, such as malaria, diarrhoea and respiratory illnesses, they were more likely to attend a health centre. Respondents described being more likely to attend traditional healers (spiritual healers and sorcerers) if they felt the response at the allopathic health services was not satisfactory. Decisions were also influenced by the household members, village head and the traditional healers. Sometimes, respondents described their reasons for visiting traditional healers as embedded in their cultural practices. Often the driving factors for following these traditions were mentioned to be rooted to their animist religion.

*I: When you were sick, where did all of you go for treatment?*

*FGD11S1: Hospital (Health centre).*

*I: Did you go to the spiritual healer (Yao)?*

*FGD11S1: Yes, I did. We always do [Yao] first before we go to hospital because this is our culture [tradition].*

*I: What about witchcraft (Mor Pao)?*

*FGD11S1: We go, if we are very sick.*

*I: The spiritual healer and witch is the same person or not?*

*FGD11S1: No! It is not same.*

FGD with eight female participants in Xuang Tai.

Community members described how their choices were often constrained by money at each stage: Respondents described how visiting spiritual healers was more frequent and more economical than visiting sorcerers who were often reserved for moderate to severe illnesses. Money, distance and a lack of appropriate transport were reported as barriers to accessing allopathic health (services) centres. The prominence of the barriers increased if respondents wanted to visit tertiary health centres (e.g. the district hospital, provincial hospital and hospital across the border). Respondents described eloquently how their subsequent visits to higher health centres if failed to cure them forced them to “return home and wait for death”.

*I: If somebody feels sick (malaria) in your village, where do they normally go for treatment??*

*FGD2S1: Danvilay health centre.*

*FGD2S2: I will buy medicine with village health volunteer.*

*FGD2S3: Danvilay health centre.*

*FGD2S5: I will treat (seek treatment) with village health volunteer if it does not work I will go to health centre.*

*FGD2S1: if the health centre does not work I will go to District hospital, if the district hospital does not work I will go to Savannakhet hospital, if Savannakhet hospital does not work I will come back home and wait to die.*

FGD with eight male participants at “Oi” sub-village of OTP.

## Discussion

This is the first study to report the treatment seeking behaviours for febrile illness in remote villages in one of the poorest district of Lao PDR with a predominantly minority *Lao Theung* population.

In remote villages of Laos, respondents highlighted several barriers to seeking treatment at allopathic health services: the absence of cash, distance to health care services, absence of transport, the availability of traditional healing practices and the expected costs of health centre visits [15, 26, 28, 29, 32, 42, 43]. Participants with low income were less likely to attend allopathic health services and this reflects the impact of income on health seeking that has been reported in other areas of Laos [26, 27, 30–34].

Cash to pay for treatment came from the sale of domestic animals, such as cows, pigs, goats, chicken and buffaloes. However, during an emergency, it was often impossible to sell domestic animals in a short period of time. The mobile merchants on whom community members depended to make a sale come at unpredictable intervals from Vietnam. This is also specific for Nong District, which borders Vietnam [24]. Economic constraints have been described as a major determinant in many low-income countries for seeking health care, including studies from Cambodia [15, 43, 44], Uganda [39] and Bangladesh [45]. Participants with lower income reported underutilization of allopathic health services, delaying the treatment seeking because often the cost of health care exceeded their monthly income which is classified as catastrophic expenditure (> 25% of monthly household income).

Proximity of the household to the health centre was found to be an important determinant of allopathic health care utilisation. In addition, factors such as lack of public transport, motorbikes, price of fuel and road conditions were associated barriers to attending health centres in Laos. Distance to health centre is a well-established determinant of care utilisation in Laos [26, 27, 29]. In Cambodia, distance to health centre was perceived as a major determinant of health service attendance [43, 44]. Similarly, in Uganda, distance to health centre was found to affect the uptake of health services [39]. In Nepal, longer distance and longer travel time were found to be associated with higher cost and recognized as a barrier for attending health centres [46]. In Nigeria, delay in attending health centre was associated with longer walking distance to the health centre [47].

Remote villages are also the hotspots for malaria transmission [3]. In such villages, allopathic health centres were popular sources of care for febrile illness, but community members’ access to these services was affected by their socio-economic status and the lack of local infrastructure. Improving essential infra-structures, particularly roads and public transportation, is needed to improve accessibility. However, the remoteness of these villages presents significant challenges to infrastructure development. Building the capacity of existing local village volunteers (village malaria workers) with additional training and strengthening their involvement with the malaria control program has been suggested by a recent study in Cambodia to mitigate barriers to accessing health services to expedite the malaria elimination [48].

As in many settings, in Nong District, respondents described how traditional healers are deeply rooted to local culture and customs [49, 50]. In the study villages, a mix of practices was described, with the preferred source of treatment influenced by illness severity, economic status and road conditions. Decisions were often influenced by the opinions of other household members, the village head, and traditional healers [12, 24, 41]. A mix of traditional and allopathic medicine has been described in past studies from Laos [51], Kenya [52] and Uganda [53]. Given their prominence and the respect that they garner in the community, malaria control programmes should consider collaborating with traditional healers as a means to strengthen their links to communities. A recent scoping review has also highlighted how peripheral health care in Laos can be strengthened by collaborating with both formal and informal health care providers for malaria elimination [8].

Amongst community members who participated in the questionnaire, more than a third of respondents were likely to seek over-the-counter medication. Popular for minor complaints, such as headache, common cold and low back pain, they are attractive options particularly because of the costs associated with visiting a district hospital or health centre. Since the 1960s [54], medicines have been purchased from authorized or unauthorized pharmacies, including, in Nong District, mobile drug vendors who visit from Vietnam [32].

### Strengths and limitation

This study used qualitative and quantitative methods for data collection. Three researchers collected data to reduce the possibility of personal bias caused by the influence of a single data collector. Cultural modesty and conformism including social desirability bias may have affected the data from FGDs and questionnaire. The findings are limited by the fact that this study took place as a baseline and formative research to inform and guide the community engagement and TME. However, we think our results are representative of the health seeking behaviour of the study population and can inform current and future malaria control programmes in Savannakhet, Lao PDR. This study derived the data from on-site translation from bilingual translators who were fluent in *Lao* and local *Lao Theung* language. The *Lao Theung* language spoken by the minority groups has no written script, some nuances and meaning could have been lost during translation. This study is also limited by the fact that health-seeking behaviour were explored in terms of febrile illnesses. Nevertheless, future studies can build on these findings to explore the overall treatment seeking behaviour for febrile illnesses, determinants of attending health services and the contribution to development of antimicrobial resistance both within Laos and outside. Respondents were asked about their past febrile illnesses in last two months and how they responded to it. Although two-months period provided respondents an adequate time to recall their febrile illnesses and pertinent past health seeking behaviour, their responses could have incurred recall bias. However, there were follow-up questions to past febrile illnesses in terms of where they sought treatment, this helped to minimize the recall errors.

## Conclusion

Treatment for febrile illness was sought from allopathic health centres and traditional practitioners, depending upon the severity of symptoms, availability of cash, distance to health centre, road conditions, and the availability of transport. In spite of these barriers, health centres were the preferred choice for treatment. Improvements in local infrastructure would probably help to overcome some of these issues but are likely to take time and considerable investment. In light of redoubled efforts to eliminate malaria, however, at least in the short-term, less costly options include strengthening the network of village health volunteers and involving traditional healers. These groups are easily accessible to community members and their roles in malaria control strategies could be strengthened through training and by formalizing their links with these programmes.

## Abbreviations

AOR: Adjusted Odds Ratio
API: Annual Parasite Index
CI: 95% Confidence Interval
FGDs: Focus Group Discussions
GMS: Greater Mekong Sub-region
LLINs: Long Lasting Insecticide Nets
MDA: Mass Drug (antimalarial) Administration
ODK: Open Data Kit
OTP: Oi Tan Tip village
PCR: Polymerase Chain Reaction
PDR: People’s Democratic Republic
PMM: Phoun Mak Mee village
TME: Targeted Malaria Elimination
TT: Tha Thay village
uPCR: ultra-sensitive Polymerase Chain Reaction
USD: United States Dollar
VMW: Village Malaria Worker
XT: Xuang Tai village
